# Generalized Robertson-Walker Space-Time Admitting Evolving Null Horizons Related to a Black Hole Event Horizon

**DOI:** 10.1155/2016/9312525

**Published:** 2016-09-18

**Authors:** K. L. Duggal

**Affiliations:** Department of Mathematics and Statistics, University of Windsor, Windsor, ON, Canada N9B 3P4

## Abstract

A new technique is used to study a family of time-dependent null horizons, called “*Evolving Null Horizons*” (ENHs), of generalized Robertson-Walker (GRW) space-time $\left(\bar{M},\bar{g}\right)$ such that the metric $\bar{g}$ satisfies a kinematic condition. This work is different from our early papers on the same issue where we used (1 + *n*)-splitting space-time but only some special subcases of GRW space-time have this formalism. Also, in contrast to previous work, we have proved that each member of ENHs is totally umbilical in $\left(\bar{M},\bar{g}\right)$. Finally, we show that there exists an ENH which is always a null horizon evolving into a black hole event horizon and suggest some open problems.

## 1. Introduction

Let $\left(\bar{M},\bar{g}\right)$ be a (1 + *n*)-dimensional space-time manifold whose metric $\bar{g}$ satisfies the following kinematic condition:

(1) $$\begin{array}{c} {\bar{\nabla }}_{X}U=\bar{\lambda }\left(\;X+\bar{g}\left(\;X,U\;\right)U\;\right), \end{array}$$

where *U* is a timelike unit vector, $\bar{\nabla }$ denotes the Levi-Civita connection on $\bar{M}$, $\bar{\lambda }$ is a function, and *X* is an arbitrary vector field on $\bar{M}$. Consider Gaussian normal coordinates (*x* ^0^ = *t*, *x* ^*a*^) on $\bar{M}$ such that *U* = ∂/∂*t* and $\bar{g}={\bar{g}}_{ij}dx^{i}dx^{j}=-dt^{2}+{\bar{g}}_{ab}dx^{a}dx^{b}$, where *i*, *j* run over 0,1,…, *n* and *a*, *b* run over 1,…, *n* and ${\bar{g}}_{ab}$ are functions of all the coordinates *x* ^*a*^. Let Σ(*t* = a constant) be a spacelike slice orthogonal to *U* and *X* = ∂/∂*x* ^*a*^ a coordinate vector field tangent to Σ. Then (1) reduces to ${\bar{\nabla }}_{\partial /\partial x^{a}}\partial /\partial t=\bar{\lambda }(\partial /\partial x^{a})$ which further implies (with some computation) that $(\partial /\partial t){\bar{g}}_{ab}=2\bar{\lambda }{\bar{g}}_{ab}$. Integrating this we obtain ${\bar{g}}_{ab}=e^{2\int \bar{\lambda }(t,x^{a})dt}{\bar{\gamma }}_{ab}$, where $\bar{\gamma }$ is a fixed Riemannian metric on the initial slice (*t* = *t*_0_ a constant). In this paper, we take $\bar{\lambda }$ as a function of *t* only and set $e^{\int \bar{\lambda }(t)dt}=\bar{\Omega }\left(t\right)$; that is, $\bar{\lambda }=\dot{\bar{\Omega }}/\bar{\Omega }$, where an overdot means derivative with respect to *t*. Then the metric $\bar{g}$ is of the form

(2) $$\begin{array}{c} \bar{g}={\bar{g}}_{ij}dx^{i}dx^{j}=-dt^{2}+{\left(\;\bar{\Omega }\;\right)}^{2}\left(\;t\;\right){\bar{\gamma }}_{ab}dx^{a}dx^{b}, \end{array}$$

which is generalized Robertson-Walker (GRW) space-time notion introduced by Alías et al. [1]. It is a warped product, with base of an open interval of a real line with a negatively defined metric and fibre of a Riemannian manifold and not of constant sectional curvature, in general. The reader will see that the kinematic condition (1) on the space-time metric plays an important role in the induced geometry of null hypersurfaces of $\bar{M}$. This is our reason to generate GRW space-time instead of stating it as a definition. This class of space-time is inhomogeneous admitting an isotropic radiation whose subcase is classical Robertson-Walker (RW) space-time which includes Einstein-de-Sitter space-time, Friedman cosmological models, and the static Einstein space-time. Considerable work is available on GRW space-time, primarily on spacelike hypersurfaces [1–4] and more cited therein, and only recently there has been interest (see Kang [5, 6] and Navarro et al. [7]) on its null submanifold geometry. However, nothing much is available on physical application of their null hypersurfaces.

It is well-known that the null hypersurfaces have been used as physical models of black hole horizons in general relativity. In this paper we show that there exists a physical model of a family of time-dependent null horizons of GRW space-time which may evolve into a black hole event horizon. See Sections 3 and 4 for a brief account on event horizons, recent works on time-dependent null horizons of Duggal [8–10] and Sultana and Dyer [11, 12], and more cited therein. We highlight that the physical use of research on time-dependent null horizons may have connection (though it is too early to be sure) with the latest LIGO [Laser Interferometer Gravitational-Waves Observatory] experiment (as per Press Release of February 11, 2016) which confirms the presence of gravitational waves produced during the final fraction of a second of the merger of two black holes into a single massive spinning black hole. Thus, this recent LIGO experiment reconfirms that black hole has a cosmological background or it is surrounded by a local matter distribution. Therefore, there is significant difference in the structure and properties of the surrounding dynamical region of a black hole and this latest experiment strengthens the ongoing research on time-dependent null horizons, in particular, near such spinning black holes surrounded with gravitational waves. For information on LIGO via video one may try http://mediaassets.caltech.edu/gwave/.

## 2. Family of Null Hypersurfaces

Consider GRW space-time $\left(\bar{M},\bar{g}\right)$ with its metric $\bar{g}$ given by (2). Let *p* be a point in $\bar{M}$ and ((*M*, *g*) : *x* ^1^ = a constant) be a timelike hypersurface of $\bar{M}$ passing through *p*. Observe that there is a freedom of choice in taking any one of the spacelike coordinates constant. Suppose that **n** is the future directed timelike unit vector on *M* induced by *U* of $\bar{M}$ such that the following holds:

(3) $$\begin{array}{c} \nabla_{X}n=\lambda \left(\;t\;\right)\left(\;X+g\left(\;X,n\;\right)n\;\right),\;\forall X\in TM, \end{array}$$

where *g*, ∇, and *λ*(*t*) are the induced metric, Levi-Civita connection, and a function on *M*, respectively. Let ((**S**, *q*) : *t*_0_ = a constant) be a connected spacelike hypersurface in *M* and hence a codimension two-spacelike submanifold of $\bar{M}$ passing through *p* via the normal exponential map along **S** in *M* with metric *q* induced from *g*. Using (3) and following a procedure explained in previous section one can show that *M* is also GRW space-time with metric *g* expressed as

(4) g=−dt2+Ω2tγABdxAdxB,2≤A,B≤n,  λ=Ω˙Ω,

where we take $\lambda =\dot{\Omega }/\Omega$ and *γ*_*AB*_ is a fixed Riemannian metric. Assume that *x* = (*x*_2_,…, *x*_*n*_) are the coordinates in (**S**_*u*_, *t* = *u* a constant) centered on *p* so that the metric *q*_*u*_ on **S**_*u*_ is given by

(5) $$\begin{array}{c} q_{u}=\Omega_{u}^{2}\gamma_{AB}dx^{A}dx^{B},\;2\leq A,B\leq n. \end{array}$$

Choose at each point of **S**_*u*_ a null direction perpendicular in **S**_*u*_ and smoothly depending on the foot-point. There are two such possibilities: the ingoing and the outgoing null directions. Then, it is easy to see that the union of all geodesics with the chosen (say outgoing) direction *ℓ*_*u*_ is a null hypersurface of $\bar{M}$. Denote by (*H*_*u*_, *h*_*u*_, *ℓ*_*u*_) such a null hypersurface of $\bar{M}$ with degenerate metric *h*_*u*_ and null normal *ℓ*_*u*_. Since *H*_*u*_ includes **S**_*u*_ and its degenerate metric *h*_*u*_ must be of signature (0, +,…, +), we assume that the Riemannian metric *q*_*u*_ of **S**_*u*_ coincides with the degenerate metric *h*_*u*_ of *H*_*u*_ given by

(6) hu=huABdxAdxB=ΩHu2γABdxAdxB,2≤A,B≤n,

where *Ω*_*H*_*u*__ is a function on *H*_*u*_ induced by the function *Ω*_*u*_ of *M*. This means that the null hypersurface (*H*_*u*_, *h*_*u*_) is conformal to a fixed null hypersurface (*H*, *γ*); that is, *h*_*u*_ = *Ω*_*H*_*u*__^2^ *γ*, with the conformal function *Ω*_*H*_*u*__.

In this paper, we take *ℓ*_*u*_ in some subset of $\bar{M}$ around *H*_*u*_. This will permit us to well define the space-time covariant derivative $\bar{\nabla }\ell_{u}$. Also, in this way we get a foliation of $\bar{M}$ (in the vicinity of *H*_*u*_) by a family (*H*_*u*_) so that *ℓ*_*u*_ is in the part of $\bar{M}$ foliated by this family (for details see Carter [13]) such that at each point in this region *ℓ*_*u*_ is a null normal to *H*_*u*_ for some value of the parameter *u*. Let (*h*_*u*_) and (*ℓ*_*u*_) be the families of degenerate metrics and null normal, respectively. Denote by

(7) $$\begin{array}{c} F=\left\{\;\left(\;\left(\;H_{u}\;\right),\left(\;h_{u}\;\right),\left(\;l_{u}\;\right)\;\right):h_{u}=\Omega_{H_{u}}^{2}\gamma \in \left(\;h_{u}\;\right)\;\right\} \end{array}$$

a family of null hypersurfaces of $\left(\bar{M},\bar{g}\right)$ conformally related to a fixed null hypersurface (*H*, *γ*, *ℓ*) and each *Ω*_*H*_*u*__ is a function on *H*_*u*_ for some value of *u*. Also, let (**S**_*u*_) be the corresponding family of submanifolds of $\bar{M}$.

The bending of each *H*_*u*_ of *F* in $\bar{M}$ is described by the* Weingarten map*:

(8) Wlu:TpHu⟶TpHu,Xu⟶∇¯Xulu.

*𝒲*_*ℓ*_*u*__ associates with each *X*_*u*_ of *H*_*u*_ the variation of *ℓ*_*u*_ along *X*_*u*_, with respect to the space-time connection $\bar{\nabla }$. The second fundamental form, say *B*_*u*_, of *H*_*u*_ is the symmetric bilinear form related to the Weingarten map by

(9) BuXu,Yu=huWluXu,Yu=hu∇¯Xulu,Y,Xu,Yu∈THu.

*B*(*X*_*u*_, *ℓ*_*u*_) = 0 for any *X*_*u*_ ∈ *TH*_*u*_ implies that *B*_*u*_ has the same *ℓ*_*u*_ degeneracy as that of the metric *h*_*u*_ and it does not depend on particular choice of *ℓ*_*u*_.

Now we deal with the question of obtaining a normalized expression for a null normal *ℓ* of a null hypersurface *H* ∈ *F*. For this purpose, we let $s\in T\bar{M}$ be a unit spacelike normal vector to **S** defined in some open neighborhood of corresponding *H*. Since the future directed timelike vectors **n** of *M* and **s** are orthogonal, it is immediate that the vector **n** + **s** is null so it is tangent to *H*. Therefore, one can take *ℓ* = **n** + **s** as a natural normalization of the null normal to *H*. However, this normalization can only be defined for a single fixed hypersurface *H*, whereas we need to normalize the entire family (*ℓ*_*u*_) for any parameter value of *u*. For this purpose, we choose the following normalization of each *ℓ*_*u*_ ∈ (*ℓ*_*u*_):

(10) lu=nu+su,where  su·su=1,  xu∈TpSu⟺su·xu=0,

which implies that each *ℓ*_*u*_ is tangent to each member of *F* and it has the property of Lie dragging the family of submanifolds (**S**_*u*_). Then, we define a transversal vector field **k**_*u*_ to *T*_*p*_ *H*_*u*_ not belonging to *F* expressed as another suitable linear combination of **n**_*u*_ and **s**_*u*_ such that it represents the ingoing direction satisfying

(11) $$\begin{array}{c} \bar{g}\left(\;l_{u},k_{u}\;\right)=-1,\;k_{u}=\frac{1}{2}\left(n_{u}-s_{u}\right). \end{array}$$

Now we are ready to state and prove the following main theorem.

**Theorem 1 .**

Let *F* = ((*H*_*u*_), (*h*_*u*_), (*ℓ*_*u*_)) be a family of null hypersurfaces of the GRW space-time $\left(\bar{M},\bar{g}\right)$ defined by (2) such that *F* is given by (7) and each of its members (*H*_*u*_, *h*_*u*_, *ℓ*_*u*_) is conformal to a fixed null hypersurface (*H*, *γ*, *ℓ*), where *h*_*u*_ = *Ω*_*H*_*u*__^2^ *γ* for every parameter value of *u*. Assume that (*H*, *γ*, *ℓ*) lies to the future of each (*H*_*u*_, *h*_*u*_). Then

(a) each (*H*_*u*_, *h*_*u*_, *ℓ*_*u*_) is totally umbilical in $\left(\bar{M},\bar{g}\right)$, with its volume expansion *θ*_(*ℓ*_*u*_)_ = (*n* − 1)*λ*_*u*_ where the function *λ*_*u*_ is as given in (3);
(b) each *ℓ*_*u*_ is conformal Killing vector (*£*_*ℓ*_*u*__ *h*_*u*_ = 2*λ*_*u*_ *h*_*u*_) on each *H*_*u*_;
(c) the family *F* may evolve into a totally geodesic null hypersurface (*H*, *γ*, *ℓ*) only if *λ*_*u*_ → 0 on *H* and then *θ*_(*ℓ*)_ = 0 on *H*.

**Proof**

Let **x**_*u*_ be a vector tangent to a spacelike hypersurface **S**_*u*_ in *M*. The Weingarten formula is given by ∇_**x**_*u*__ **n**_*u*_ = *A*_**n**_*u*__ **x**_*u*_, where we denote by *A*_**n**_*u*__ the shape operator of **S**_*u*_. It follows from (3) that *A*_**n**_*u*__ **x**_*u*_ = *λ*_*u*_ **x**_*u*_. Denote by *B*_**S**_*u*__ the second fundamental form of **S**_*u*_ in *M* with respect to **n**_*u*_. Then, for any **x**_*u*_, **y**_*u*_ ∈ *TS*_*u*_

(12) BSuxu,yuqu∇xunu,yu=quAnuxu,yu=λuquxu,yu.

Thus, (**S**_*u*_, *q*_*u*_) is totally umbilical in *M*. Then, it is easy to show that any (*H*_*u*_, *h*_*u*_, *ℓ*_*u*_) is also totally umbilical in $\bar{M}$. Indeed, take *B*_*u*_ as the second fundamental form of *H*_*u*_. As *B*_*u*_(*ℓ*_*u*_, *X*_*u*_) = 0, ∀*X*_*u*_ ∈ *TH*_*u*_, and corresponding **S**_*u*_ ⊂ *H*_*u*_ we conclude that for every *X*_*u*_, *Y*_*u*_ ∈ *TH*_*u*_

(13) BuXu,Yu=Buxu,yu=BSuxu,yu,∀xu,yu∈TSu.

Since the Riemannian metric *q*_*u*_ coincides with the corresponding degenerate metric *h*_*u*_ it follows from above relation and (12) that

(14) $$\begin{array}{c} B_{u}\left(\;X_{u},Y_{u}\;\right)=\lambda_{u}h_{u}\left(\;X,Y\;\right),\;\forall X_{u},Y_{u}\in TH_{u}, \end{array}$$

which proves that each *H*_*u*_ is totally umbilical in $\bar{M}$. Moreover, it follows from (3) that the volume expansion *θ*_(*ℓ*_*u*_)_ = div⁡(**n**_*u*_) = (*n* − 1)*λ*_*u*_, where we have used *B*_*u*_(*ℓ*_*u*_, *X*_*u*_) = 0, ∀*X*_*u*_ ∈ *TH*_*u*_, which proves (a). Using the expression

(15) $$\begin{array}{c} {£}_{l_{u}}h_{u}\left(\;X,Y\;\right)=h_{u}\left(\;{\bar{\nabla }}_{X}l_{u},Y\;\right)+h_{u}\left(\;{\bar{\nabla }}_{Y}l_{u},X\;\right) \end{array}$$

and *B*_*u*_(*X*_*u*_, *Y*_*u*_) symmetric in the above equation we obtain

(16) $$\begin{array}{c} B_{u}\left(\;X_{u},Y_{u}\;\right)=\frac{1}{2}{£}_{l}h_{u}\left(\;X_{u},Y_{u}\;\right),\;\forall X_{u},Y_{u}\in TH_{u}. \end{array}$$

Since *H*_*u*_ is totally umbilical, we use *B*_*u*_(*X*_*u*_, *Y*_*u*_) = *λ*_*u*_ *h*_*u*_(*X*_*u*_, *Y*_*u*_) in above relation to get *£*_*ℓ*_*u*__ *h*_*u*_ = 2*λ*_*u*_ *h*_*u*_ on *H*_*u*_. Hence, each *ℓ*_*u*_ is conformal Killing vector of the metric *h*_*u*_ with conformal function 2*λ*_*u*_ which proves (b). For item (c), since the fixed hypersurface (*M*, *γ*, *ℓ*) is in the future of its conformally related family *F* of hypersurfaces, (*H*_*u*_, *h*_*u*_)→(*H*, *γ*) only if the metric *h*_*u*_ → *γ* for that value of *u*, which further means that *Ω*_*u*_ → 1 for that value of *u*. We know that $\lambda_{u}=\dot{\Omega_{u}}/\Omega_{u}$. Thus, *λ*_*u*_ → 0 on *H* for that value of *u* which implies that *θ*_(*ℓ*)_ = 0 on *H*. Now we know that a null hypersurface of a semi-Riemannian manifold has zero expansion if and only if it is totally geodesic so (c) holds which completes the proof.

Now we quote the following general result for a totally umbilical submanifold (also holds for totally geodesic case) of a semi-Riemannian manifold which is needed to address the question of how Theorem 1 can be used to show the existence of null horizons of GRW space-time.

**Proposition 2 (Perlick [14]).**

Let *H* be a totally umbilical submanifold of a semi-Riemannian manifold $\bar{M}$. Then, (a) a null geodesic vector field of $\bar{M}$ that starts tangential to *H* remains within *H* (for some parameter interval around the starting point) and (b) *H* is totally geodesic if and only if every geodesic vector field of $\bar{M}$ that starts tangential to *H* remains within *H* (for some parameter interval around the starting point).

Above result satisfies a requirement for the existence of a null horizon in relativity. Since we assume that each null normal *ℓ*_*u*_ of the family of hypersurfaces *F* is null geodesic, using above result of Perlick, we state the following corollary of Theorem 1 (proof is easy).

**Corollary 3 .**

Let $\bar{(M},\bar{g})$ be null geodesically complete space-time obeying the null energy condition $\bar{Ric}(X,X)\geq 0$ for all null vectors *X* such that the hypothesis of Theorem 1 holds. Then, each null geodesic vector *ℓ*_*u*_ of *F* is contained in its respective smooth totally umbilical null hypersurface (*H*_*u*_, *ℓ*_*u*_) of $\left(\bar{M},\bar{g}\right)$. In particular, this property will also hold for the totally geodesic null hypersurface (*H*, *γ*) of $\left(\bar{M},\bar{g}\right)$.

## 3. Physical Interpretation

In this section we present a physical interpretation of Theorem 1. For this purpose, recall that the vorticity-free Raychaudhuri equation for any member (*H*, *h*, *ℓ*) of the family *F* is given by

(17) $$\begin{array}{c} \frac{d\left(\theta_{\left(l\right)}\right)}{ds}=-{\bar{R}}_{ij}l^{i}l^{j}-\sigma_{ij}\sigma^{ij}-\frac{\theta^{2}}{n-1}, \end{array}$$

where $\sigma_{ij}=\overset{-}{\underset{\leftarrow }{\nabla }}{\;}_{(i}{\left(\ell \right)}_{j)}-(1/2)\theta_{\left(\ell \right)}h_{ij}$ is the shear tensor, *s* is a pseudoarc parameter such that *ℓ* is null geodesic, and ${\bar{R}}_{ij}$ is the Ricci tensor of $\bar{M}$. We also recall that in two recent papers [8, 9] we studied a new class of null horizons of space-time using the following definition.

**Definition 4 .**

A null hypersurface (*H*, *h*, *ℓ*) of the family *F* of space-time $\left(\bar{M},\bar{g}\right)$ is called an* Evolving Null Horizon*, briefly denoted by ENH, if

(i) *H* is totally umbilical in $\bar{M}$ and may include a totally geodesic portion;
(ii) all equations of motion hold at *H* and energy tensor *T*_*ij*_ is such that *T*_*b*_^*a*^ *ℓ* ^*b*^ is future-causal for any future directed null normal *ℓ*.

Comparing this definition with Theorem 1, we notice that the first part of its condition (i) is the same as the first part of conclusion (a) of Theorem 1 and energy condition (ii) requires that ${\bar{R}}_{ij}\ell^{i}\ell^{j}$ is nonnegative for any *ℓ*, which implies (see Hawking and Ellis [15, page 95]) that *θ*_(*ℓ*)_ monotonically decreases in time (which we take as *u* parameter for the family *F*) along each *ℓ*; that is, $\bar{M}$ obeys the null convergence condition. Thus, in the region where *θ*_(*ℓ*)_ is nonzero each member of the family *F* will be totally umbilical with *u*-parameter time-dependent metric *h* and may include a totally geodesic portion where *θ*_(*ℓ*)_ vanishes for a fixed (*H*, *γ*), so condition (c) will hold. It follows from the Raychaudhuri equation that each *ℓ* will have nonzero or zero shear accordingly as its expansion function *θ*_(*ℓ*)_ is nonzero or zero, respectively. Moreover, it is easy to see that condition (b) of Theorem 1 will hold for an ENH. This clearly shows that there exists a “*Physical Model*” of a class *F* = ((*H*_*u*_), (*h*_*u*_), (*ℓ*_*u*_)) of a family of totally umbilical null hypersurfaces of the space-time $\left(\bar{M},\bar{g}\right)$, satisfying the hypothesis and three conclusions of Theorem 1, such that each of its members is an Evolving Null Horizon (ENH) which may evolve into a fixed null hypersurface (*H*, *γ*) with zero expansion, *θ*_(*ℓ*)_ = 0. For up-to-date information with some examples of ENHs we refer to Duggal [8–10, 16].

However, Theorem 1 is limited by the fact that not every such totally umbilical null hypersurface evolving into a totally geodesic null hypersurface can be related to a black hole horizon. Therefore, we must show that there exists a prescribed family of ENHs of GRW space-time which is related to a black hole horizon. To complete our analysis on a physical interpretation we need the following information on a class of black hole horizons.

*Event Horizons*. A boundary of space-time is called a null* event horizon* (briefly denoted by EH) beyond which events cannot affect the observer. An EH is intrinsically a global concept as its definition requires the knowledge of the entire space-time to determine whether null geodesics can reach null infinity. In particular, an event horizon is called a Killing horizon if it is represented by a null hypersurface which admits a Killing vector field. Most important family is the Kerr-Newman black holes. EHs have played a key role and this includes Hawking's area increasing theorem, black hole thermodynamics, black hole perturbation theory, and the topological censorship results. Moreover, an EH always exists in black hole asymptotically flat space-time under a weak cosmic censorship condition and is represented by a Killing horizon such that the space-time is analytic and the stress tensor obeys the weak energy condition. The null hypersurface of such space-time admits a nonvanishing Killing vector field, say *ℓ*, which may or may not be the Killing vector field of the landing space-time. The latter case corresponds to a rotating asymptotically flat black hole which we do not discuss here. We refer to Hawking's paper on “event horizons” [17], three papers of Hájiček's work [18–20], and more cited therein. Based on this brief account, we now recall the following work of Galloway [21] who has shown that the null hypersurfaces which arise most naturally in general relativity, such as black hole EHs, are in general *C* ^0^ but not *C* ^1^. His approach has its roots in the well-known geometric maximum principle of E. Hopf, a powerful analytic tool which is often used in the theory of minimal or constant mean curvature hypersurfaces. This principle implies that two different minimal hypersurfaces in a Riemannian manifold cannot touch each other from one side. In year 2000, Galloway [21] proved the following result for smooth null hypersurfaces restricted to the zero mean curvature case and suitable for asymptotically flat space-time.

**Theorem 5 .**

Let *H*_1_ and *H*_2_ be smooth null hypersurfaces in a space-time manifold $\bar{M}$. Suppose that (1)  *H*_1_ and *H*_2_ meet at $p\in \bar{M}$ and *H*_2_ lies to the future side of *H*_1_ near *p* and (2) the null mean curvatures *θ*_1_ of *H*_1_ and *θ*_2_ of *H*_2_ satisfy *θ*_2_ ≤ 0 ≤ *θ*_1_. Then *H*_1_ and *H*_2_ coincide near *p* and this common null hypersurface has mean curvature *θ* = 0.

Although the above maximum principle theorem is for smooth null hypersurfaces, in reality null hypersurfaces as models of black hole event horizons are the null portions of achronal boundaries as the sets $=\partial I^{\pm }(A),\;\;A\subset \bar{M}$, which are always *C* ^0^ hypersurfaces and contain nondifferentiable points. These null geodesics (entirely contained in *C* ^0^ null hypersurface *H*) are the null geodesic generators of *H*. For this reason, Galloway also proved his above result for *C* ^0^ null hypersurface and the following physically meaningful* null splitting theorem*.

**Theorem 6 (see [21]).**

Let $\left(\bar{M},\bar{g}\right)$ be null geodesically complete space-time which obeys the null energy condition, $\bar{Ric}(X,X)\geq 0$ for all null vectors *X*. If $\bar{M}$ admits a null line *η*, then *η* is contained in a smooth closed achronal totally geodesic null hypersurface.

Examples (see [21]) are Minkowski space, de-Sitter, and anti-de-Sitter spaces. Now we state the following theorem as physical interpretation of Theorem 1.

**Theorem 7 .**

Let $\left(\bar{M},\bar{g}\right)$ be null geodesically complete GRW space-time which obeys the null energy condition, $\bar{Ric}(X,X)\geq 0$ for all null vectors *X*. Suppose *ℱ* = ((*H*_*u*_), (*h*_*u*_), (*ℓ*_*u*_)) is a family of null hypersurfaces of $\left(\bar{M},\bar{g}\right)$ defined by (2) and given by (7) where each of its members (*H*_*u*_, *h*_*u*_, *ℓ*_*u*_) is conformally related to a totally geodesic hypersurface (*H*, *γ*, *ℓ*) such that the conformal factor of *h*_*u*_ = *Ω*_*H*_*u*__^2^ *γ* for every parameter value of *u*. Then, conditions (a) and (b) of Theorem 1 will hold. Moreover, let (*H*, *γ*, *ℓ*) be in the future of each (*H*_*u*_, *h*_*u*_, *ℓ*_*u*_) and it admits a Killing horizon. Then, (c) the family *ℱ* evolves into a black hole event horizon represented by (*H*, *γ*, *ℓ*) only if *λ*_*u*_ → 0 at null infinity.

The proofs of items (a) and (b) follow exactly as the proofs of items (a) and (b) in Theorem 1. For the proof of item (c) we first notice that *H* admits a Killing horizon. Secondly, the Killing horizon *H* satisfies a particular case of Corollary 3 and Galloway's [21] null splitting Theorem 6. This implies that when *λ*_*u*_ → 0 the Killing horizon *H* will be equivalent to an event horizon at null infinity, which proves (c).

We, therefore, claim that Theorem 1 provides a physical model of a family of time-dependent Evolving Null Horizons (ENHs) of GRW space-time and Theorem 7 is a step towards the ongoing physical use of ENHs of prescribed GRW space-time which admits a null event horizon satisfying Galloway's null splitting Theorem 6 whose above listed three examples (which are subcases of GRW space-time) are also examples of Theorem 7. Consequently, the two Theorems 1 and 7 complete the objective of this paper.

## 4. Conclusion

The motivation for this work originated from the need for time-dependent null horizons which describe the geometry of a null hypersurface of dynamical space-time. For this purpose, in our four previous papers [8–10, 16] we studied a quasilocal family of “*Evolving Null Horizons*” (ENHs) of (1 + *n*)-splitting space-time, explained the reason for such a study, constructed a variety of examples of ENHs, and in some cases established their relation with black hole isolated horizons introduced by Ashtekar et al. [22]. The following is a brief account on nonexpanding, weakly, and isolated horizons.

**Definition 8 .**

A null hypersurface (*H*, *γ*) of 4-dimensional space-time $\left(\bar{M},\bar{g}\right)$ is called a* nonexpanding horizon* (NEH) if (1)  *H* has a topology *R* × *S* ^2^, (2) any null normal *ℓ* ^*a*^ of *H* has vanishing expansion, *θ*_(*ℓ*)_ = 0, and (3) all equations of motion hold at *H* and the stress energy tensor *T*_*ij*_ is such that −*T*_*j*_^*i*^ *ℓ* ^*j*^ is future-causal for any future directed null normal *ℓ* ^*i*^.

Condition (1) implies that *H* is ruled by the integral curves of the null direction field which is normal to it. Conditions (2) and (3) imply that *£*_*ℓ*_ *γ* = 0 on *H*, which further implies that the metric *γ* is time-independent and *H* is totally geodesic in $\bar{M}$. Also, it follows from the Raychaudhuri equation that *ℓ* is shear-free on *H*. In general, there does not exist a unique induced connection on *H* due to degenerate *γ*. However, on an NEH, the property *£*_*ℓ*_ *γ* = 0 implies that the space-time connection $\bar{\nabla }$ induces a unique (torsion-free) connection, say *𝒟*, on *H* compatible with *γ*. We say that two types of null normal, *ℓ* and $\bar{\ell }$, belong to the same equivalence class [*ℓ*] if $\ell =c\bar{\ell }$ for some positive constant *c*.

**Definition 9 .**

The pair (*H*, [*ℓ*]) is called a weakly isolated horizon (WIH) if it is NEH and each normal *ℓ* ∈ [*ℓ*] satisfies (*£*_*ℓ*_ *𝒟*_*i*_ − *𝒟*_*i*_ *£*_*ℓ*_)*ℓ* ^*i*^ = 0; that is, *𝒟*_*i*_ *ℓ* ^*j*^ is time-independent. Moreover, WIH (*H*, *γ*, [*ℓ*]) is called an* isolated horizon* (IH) if the full connection *𝒟* is time-independent, that is, if (*£*_*ℓ*_ *𝒟*_*i*_ − *𝒟*_*i*_ *£*_*ℓ*_)*V* = 0 for arbitrary vector fields *V* tangent to *H*.

We refer to Ashtekar et al. [23] and several other papers listed therein for information with examples on isolated horizons.

In this paper, for the first time in the literature we have studied the existence of a family of time-dependent ENHs in GRW space-time which, in general, is not (1 + *n*)-splitting space-time. Since our early works in which the study on ENHs of (1 + *n*)-splitting space-time was explored, we have used a new technique of using the kinematic condition (1) for constructing the GRW space-time (instead of defining it) and proving Theorems 1 and 7 is an important step towards the ongoing research on time-dependent null horizons. On the other hand we mention that recently Caballero et al. [4] have proved the following characterization of prescribed space-time which can be globally split as GRW space-time.

**Theorem 10 .**

A Lorentzian manifold $\left(\bar{M},\bar{g}\right)$ admits a global decomposition as GRW space-time if and only if it has a timelike gradient conformal Killing vector field *K*, such that the flow of its normalized vector field, *Z*, is well defined and onto a domain *I* × *ℒ* for some interval *I* ≤ *R* and some leaf of the orthogonal foliation to *K*.

Contrary to this, note that our working GRW space-time does not require a conformal Killing vector field. Also, observe that in this paper we have only related ENHs to black hole event horizons represented by a Killing horizon, whereas in our previous works [8–10, 16] we related ENHs to one of the three types of isolated horizons which, unlike event horizons, may not be represented by a Killing horizon. Note however that any Killing horizon which is topologically *R* × *S* ^2^ is an isolated horizon which is the only common result between this paper and our previous papers on the same issue. It is important to mention that Lewandowski [24] has shown that Einstein's equations admit an infinite dimensional family of solutions with isolated horizons which are not Killing horizons. A subfamily of Robertson-Trautman space-time provides explicit examples of space-time which admit IHs but do not admit a Killing vector in any neighborhood of it [25].

Finally, we discuss similarity and difference between our results in this paper and two papers of Sultana and Dyer [11, 12] related to common issue of existence of time-dependent null horizons. In [11], they considered a conformal transformation $\bar{g}=\Omega^{2}g$ to a stationary, asymptotically flat space-time (*M*, *g*) admitting a Killing horizon *H*. They have shown that such a hypersurface *H* is null geodesic, called a conformal Killing horizon (CKH) if and only if the twist of the conformal Killing trajectories on *M* vanishes. Moreover, CKHs are time-dependent and they have a link with an event horizon. In [12] they constructed an example of CKH in de-Sitter space-time. Consequently, although their result on existence of time-dependent CKH is similar to the conclusions of Theorems 1 and 7, their work is only limited to null hypersurfaces of asymptotically flat space-time whereas our results in this paper are applicable to a variety of GRW space-time.

## 5. Future Prospects

- Since an event horizon refers to infinity, it is more appropriate (see details in [23]) to use one of the three types of isolated horizons which can easily locate a black hole. For this reason, one may try different approach to show the existence of a family of ENHs of a prescribed class of GRW space-time which relate to isolated horizons. This opens the possibility of using the global splitting Theorem 10 and then proving a result similar to Theorem 1 for existence of ENHs related to an isolated horizon.
- We know that an event horizon always exists in black hole asymptotically flat space-time and isolated horizons approximate event horizons of a black hole at late stages of gravitational collapse and can be easily located, as stated in our previous papers. However, the following question still remains open:* Does there always exist an ENH of black hole space-time?*
- Since an ENH comes from a family of null hypersurfaces, its existence and uniqueness are questionable, which is still an open problem. We need an input from interested readers for these open problems.
